# Monoamine Oxidase Inhibitors Prevent Glucose-Dependent Energy Production, Proliferation and Migration of Bladder Carcinoma Cells

**DOI:** 10.3390/ijms231911747

**Published:** 2022-10-04

**Authors:** Jessica Resta, Yohan Santin, Mathieu Roumiguié, Elodie Riant, Alexandre Lucas, Bettina Couderc, Claudia Binda, Philippe Lluel, Angelo Parini, Jeanne Mialet-Perez

**Affiliations:** 1 Institute of Metabolic and Cardiovascular Diseases (I2MC), INSERM, Toulouse University, 31000 Toulouse, France; 2 Department of Urology, CHU-Institut Universitaire du Cancer de Toulouse, 31000 Toulouse, France; 3 Centre de Recherches en Cancérologie de Toulouse (CRCT), INSERM, Toulouse University, 31000 Toulouse, France; 4 Department of Biology and Biotechnology, University of Pavia, 27100 Pavia, Italy; 5 Urosphere SAS, 3 rue des Satellites, 31400 Toulouse, France

**Keywords:** monoamine oxidases, tumorigenesis, cancer, oxidative stress, glycolysis, glucose transport

## Abstract

Bladder cancer is the 10th most common cancer in the world and has a high risk of recurrence and metastasis. In order to sustain high energetic needs, cancer cells undergo complex metabolic adaptations, such as a switch toward aerobic glycolysis, that can be exploited therapeutically. Reactive oxygen species (ROS) act as key regulators of cancer metabolic reprogramming and tumorigenesis, but the sources of ROS remain unidentified. Monoamine oxidases (MAOs) are mitochondrial enzymes that generate H_2_O_2_ during the breakdown of catecholamines and serotonin. These enzymes are particularly important in neurological disorders, but recently, a new link between MAOs and cancer has been uncovered, involving their production of ROS. At present, the putative role of MAOs in bladder cancer has never been evaluated. We observed that human urothelial tumor explants and the bladder cancer cell line AY27 expressed both MAO-A and MAO-B isoforms. Selective inhibition of MAO-A or MAO-B limited mitochondrial ROS accumulation, cell cycle progression and proliferation of bladder cancer cells, while only MAO-A inhibition prevented cell motility. To test whether ROS contributed to MAO-induced tumorigenesis, we used a mutated form of MAO-A which was unable to produce H_2_O_2_. Adenoviral transduction of the WT MAO-A stimulated the proliferation and migration of AY27 cells while the Lys305Met MAO-A mutant was inactive. This was consistent with the fact that the antioxidant Trolox strongly impaired proliferation and cell cycle progression. Most interestingly, AY27 cells were highly dependent on glucose metabolism to sustain their growth, and MAO inhibitors potently reduced glycolysis and oxidative phosphorylation, due to pyruvate depletion. Accordingly, MAO inhibitors decreased the expression of proteins involved in glucose transport (GLUT1) and transformation (HK2). In conclusion, urothelial cancer cells are characterized by a metabolic shift toward glucose-dependent metabolism, which is important for cell growth and is under the regulation of MAO-dependent oxidative stress.

## 1. Introduction

Urothelial cell carcinoma (UCC) accounts for about 90% of all bladder cancers and is the 10th most common cancer in the world. A constant increase in the incidence of bladder cancer is observed due to the aging of the population and tobacco smoking [1]. Bladder cancers are classified as non-muscle-invasive (NMIBC) or muscle-invasive (MIBC) and, while localized forms of urothelial cancer carry an excellent prognosis, invasion of muscle layers is associated with poor recovery and survival. Despite improvement in early diagnosis and treatment mostly based on surgery and immunotherapy, bladder cancer remains a significant contributor to cancer burden worldwide [2]. At present, efforts to deeply understand the pathogenic mechanisms that support urothelial carcinogenesis could help in identifying new therapeutic targets.

Monoamine oxidases (MAOs) have been described as important determinants in age-associated disorders such as neurodegenerative and chronic diseases, and more recently cancers [3]. The two isoforms MAO-A and MAO-B are mitochondrial flavoenzymes involved in the metabolism of neurotransmitters such as norepinephrine, dopamine and serotonin that generate substantial amounts of hydrogen peroxide (H_2_O_2_) during oxidative deamination of substrates [4]. MAOs play important roles in the central nervous system by regulating neurotransmitter levels [5]. They are also strongly expressed in peripheral tissues such as the kidney and heart, where they are at the origin of pathological processes (renal and heart failure, senescence/accelerated aging) in relation to their production of reactive oxygen species (ROS) [6,7,8,9]. Intriguingly, MAOs have recently been demonstrated to be upregulated in different types of cancer such as lung cancer and glioblastoma [10,11]. In addition, MAO-A expression correlates with the stage of prostate tumorigenesis [5]. Thus, the implication of MAOs in central and peripheral diseases has prompted the development of new reversible and selective inhibitors, some of them being currently used to treat Parkinson’s disease such as safinamide for MAO-B [4]. On the other hand, selective MAO-A inhibitors are of great interest in counteracting the growth of prostate cancer cells and metastasis through the inhibition of ROS-driven pro-tumorigenic signaling pathways [12,13].

The role of ROS in carcinogenesis is intricate, and cancer cells can increase their rate of ROS production together with their antioxidant capacities to maintain ROS homeostasis (ROS signaling) and escape cell death [14,15]. Concerning urothelial cancer cells, mounting evidence demonstrates that ROS regulate both the initiation and the progression of tumorigenesis by activating proliferative, angiogenic and pro-survival pathways such as MAPK, NRF2, PI3K/Akt and HIF-1α [16]. These pathways are also responsible for the reprogramming of energy metabolism that occurs in many cancer cells [17]. The enhanced conversion of glucose to lactate in the presence of oxygen is a crucial component of the malignant phenotype, according to the “Warburg theory” [18]. It is believed to sustain the growth and proliferation of cancer cells by rapidly providing intermediate metabolites for amino acid and nucleotide synthesis [19]. This high rate of glycolysis, together with the high output of lactate and extracellular acidification, offers a growth advantage to tumor cells and leads to a more aggressive tumorigenic phenotype that could be exploited for treatment. From a mechanistic point of view, metabolic reprogramming is associated with the upregulation of glucose transporter (Glut1) and different enzymes involved in glycolysis (hexokinase 2 (HK2), the low-activity pyruvate kinase M2 (PKM2) and the strongly overexpressed lactate dehydrogenase A (LDHA)) [18]. Whether mitochondrial dysfunction is at the origin of the metabolic switch in cancer still remains unclear, and oxidative phosphorylation (OXPHOS) can be preserved, conferring some metabolic flexibility in cancer cells [20]. At present, the specific sources of ROS and their mechanisms of action in metabolic reprogramming, proliferation and migration of bladder cancer cells remain largely unknown. Thus, we aimed at elucidating the biological role played by MAO-A and MAO-B in bladder cancer tumorigenesis, with respect to ROS production and metabolic adaptation.

Here, we show that human bladder cancer explants express both MAO-A and MAO-B isoenzymes and that pharmacological blockade of MAOs hinders mitochondrial ROS burden, proliferation and motility of AY27 bladder cancer cells. We also show that MAO-dependent ROS production is necessary for tumorigenesis as overexpression of a MAO-A mutant defective in H_2_O_2_ generation lacks any effect on cell proliferation and motility compared to WT MAO-A. Finally, we observe that MAO activation is indeed a strong inducer of glucose metabolism through the upregulation of Glut1 and HKII enzymes in cancer cells, favoring proliferation.

## 2. Results

### 2.1. Expression and Activity of MAOs in Human Bladder Tumors and Rat Carcinoma Cells

**Figure 1:**
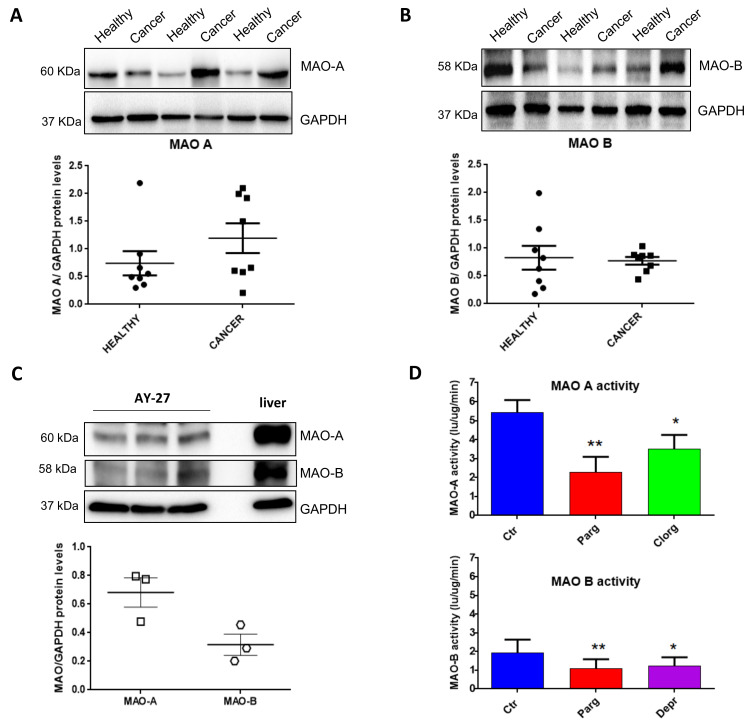
Expression of MAOs in human bladder tumors and AY27 cells. (**A**,**B**) Protein expression of MAO-A and MAO-B in human bladder tissues (health vs. cancer), N = 8. (**C**) Protein expression of MAO-A and MAO-B in AY27 cells, N = 3. (**D**) Quantification of MAO-A and MAO-B activities in AY27 cells in control conditions (Ctr) or after inhibitor treatment with pargyline 10^−5^ M, clorgyline 10^−6^ M or deprenyl 10^−6^ M (N = 5). * *p* < 0.05, ** *p* < 0.01 compared with Ctr cells. All the values are expressed as mean ± SEM.

In order to determine the potential implication of MAOs in the onset/progression of cancer phenotype, we examined the expression of MAOs in resected tissues from human bladder tumors compared to the healthy bladder tissues of the same patients. Immunoblot analysis showed that MAO-A and MAO-B were both expressed in human bladders (Figure 1A,B). Between healthy and tumor samples, there was a trend toward increased MAO-A expression in tumor samples, but not MAO-B (Figure 1A,B). We next evaluated the expression of MAO isoenzymes in a well-known bladder cancer rat cell line, AY27. We observed that both MAO-A and MAO-B were expressed in these cells, with a higher ratio of MAO-A to GAPDH as compared to MAO-B (Figure 1C). As expected, MAO-A and MAO-B activities were inhibited by the non-selective compound pargyline (Figure 1D). In addition, MAO-A activity was reduced with the selective inhibitor clorgyline, while MAO-B activity was inhibited by deprenyl.

### 2.2. Effect of MAO Inhibition on the Tumor Phenotype of Urothelial Cells

**Figure 2:**
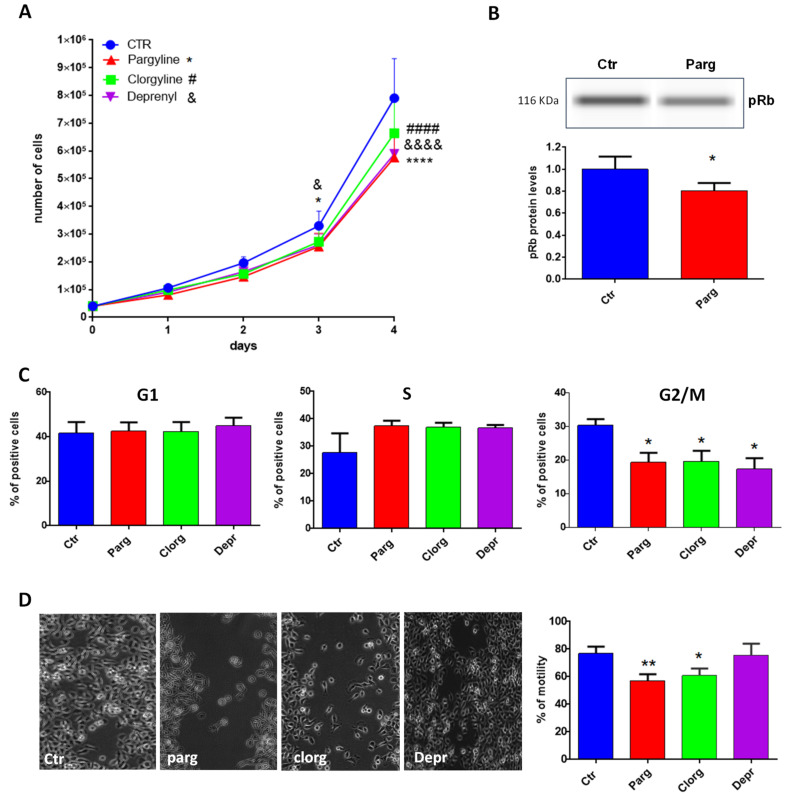
MAO effects on proliferation and motility. (**A**) Proliferation curve of AY27 cells cultured in standard media supplemented or not with different inhibitors (pargyline 10^−5^ M, clorgyline 10^−6^ M, deprenyl 10^−6^ M). The number of cells was compared to control condition from day 1 until day 4, N = 3. (**B**) The expression of phospho-Rb was measured by WES in AY27 cells treated or not with pargyline 10^−5^ M. N = 8. (**C**) The number of AY27 cells in each phase of the cell cycle was examined by flow cytometric analysis with propidium iodide DNA staining. N = 5. (**D**) AY27 cells cultured in standard media supplemented or not supplemented with different inhibitors (pargyline 10^−5^ M, clorgyline 10^−6^ M, deprenyl 10^−6^ M), were wounded and then re-treated for 24 h. Quantification of images was performed at 24 h. N = 6. * *p* < 0.05, ** *p* < 0.01, **** *p* < 0.0001, #### *p* < 0.0001, &&&& *p* < 0.0001 compared with Ctr cells. All the values are expressed as mean ± SEM.

We next determined the effects of MAO inhibition on the pro- or anti-tumor phenotype of AY27 cells by measuring proliferation, one of the main characteristics of cancer cells. As shown in Figure 2A, treatment with the non-selective MAO inhibitor pargyline significantly inhibited the growth of AY27 cells, compared to control. In order to assess the specific role played by MAO-A or MAO-B, we used selective MAO-A (clorgyline) or MAO-B (deprenyl) inhibitors. Interestingly, both inhibitors reduced the number of AY27 cells after 4 days in culture, indicating that MAO-A and MAO-B may have redundant effects (Figure 2A). In order to determine whether the inhibition of cell growth was accompanied by a decrease in cell viability, we evaluated the proportion of apoptotic and necrotic cells by analyzing phosphatidyl serine externalization and membrane permeability with Annexin-V-FITC/propidium iodide labeling. Figure S1 shows that incubation of AY27 cells with pargyline did not promote apoptosis (left panel) or necrosis of AY27 cells (right panel).

We next measured cell cycle markers in order to better understand the effects of MAO inhibition on AY27 cells. The retinoblastoma tumor suppressor protein Rb regulates cell proliferation by controlling progression through the restriction point within the G1 phase of the cell cycle. Retinoblastoma protein (Rb) in a hypo-phosphorylated state prevents the progression of the cell cycle. Incubation of AY27 cells with pargyline for 72 h induced a significant decrease in the levels of phospho-Rb (Figure 2B). We also measured the progression of the cell cycle by flow cytometry in the presence of MAO inhibitors. Incubation with MAO-A or MAO-B specific inhibitors or with the non-selective inhibitor pargyline induced a significant decrease in the percentage of cells in the G2/M phase after 72 h of treatment, indicating a block in the S phase (Figure 2C). A permanent exit from the cell cycle can be associated with senescence, which is a potent anti-tumor cellular response. Indeed, we found that pargyline increased the number of senescence-associated SA-βgal-positive cells at 96 h, indicating a block in proliferation due to the induction of senescence (Figure S2).

Tumor cell mobility plays a key role in invasion and metastasis. Therefore, we tested the effects of MAO inhibitors on the motility of AY27 cells by wound healing assay. The cell layer was scratched with a micropipette tip to generate a wound, and videomicroscopy was used to record images every 30 min for 48 h. As shown in Figure 2D, pargyline and clorgyline significantly inhibited the motility of AY27 cells, while deprenyl had no effect.

**Figure 3:**
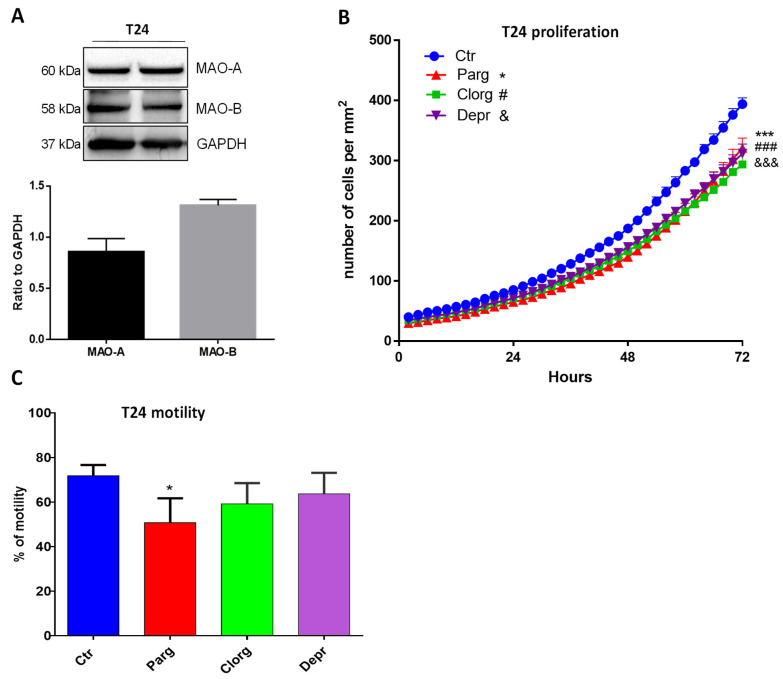
MAO inhibitor effects on proliferation and motility of human T24 cells. (**A**) Protein expression of MAO-A and MAO-B in T24 cells, N = 3. (**B**) Proliferation curve of T24 cells cultured in standard media supplemented or not with different inhibitors (pargyline 10^−5^ M, clorgyline 10^−6^ M, deprenyl 10^−6^ M). The number of cells was automatically counted every 2 h (Incucyte, Sartorius) and compared to Ctrl condition on day 3, N = 4. (**C**) T24 cells cultured in standard media supplemented or not supplemented with different inhibitors (pargyline 10^−5^ M, clorgyline 10^−6^ M, deprenyl 10^−6^ M), were wounded and then re-treated for 24 h. Quantification of images was performed at 24 h. N = 4. * *p* < 0.05, *** *p* < 0.001, ### *p* < 0.001, &&& *p* < 0.001 compared with Ctr cells. All the values are expressed as mean ± SEM.

We next wondered whether the effects that we observed also occurred in human bladder cancer cells. We used the T24 cell line, derived from a human malignant bladder cancer. As shown in Figure 3A, T24 cells expressed both MAO-A and MAO-B proteins. In addition, inhibition of either MAO-A (clorgyline) or MAO-B (deprenyl) enzymes, or both with pargyline, decreased cell proliferation (Figure 3B). On the other hand, while pargyline decreased cell motility of T24, clorgyline showed only a tendency while deprenyl had no effect (Figure 3C).

In conclusion, while both MAO-A and MAO-B regulate the proliferation of different bladder cancer cells, only MAO-A promotes the motility of these cells.

### 2.3. Effect of MAO-Derived Oxidative Stress on Proliferation and Migration of AY27 Cells

**Figure 4:**
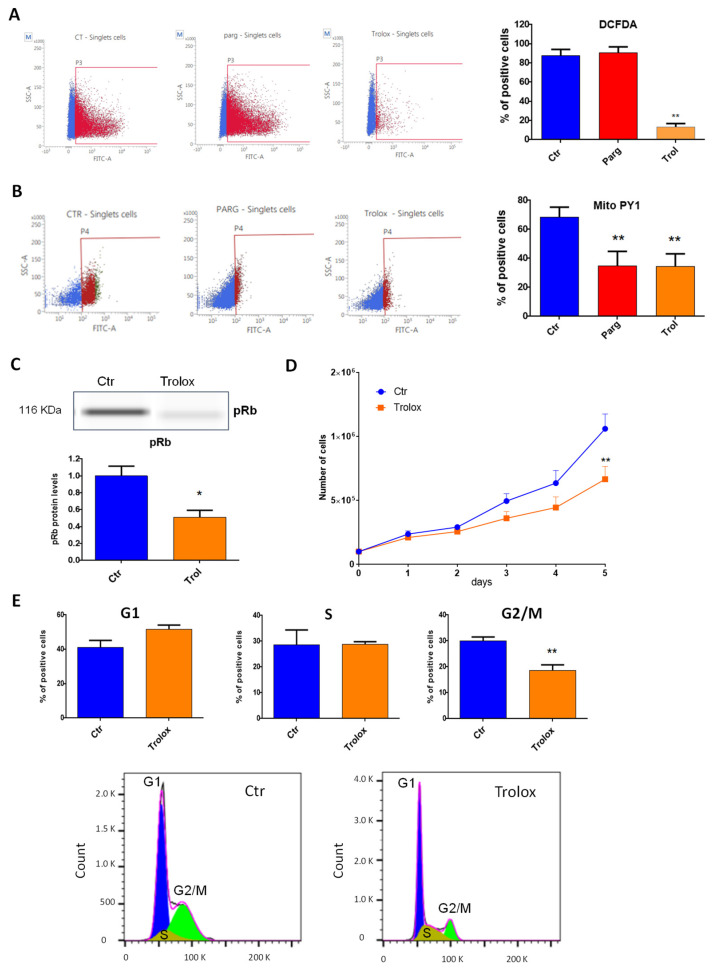
ROS content and role in AY27 cells. (**A**,**B**) ROS content was measured with either the DCFDA probe (2′,7′_dichlorofluorescin diacetate) (N = 3) (**A**) or the MitoPY1 probe (Mitochondria Peroxy Yellow 1) (N = 4) (B) by flow cytometry in AY27 cells treated or not with pargyline or Trolox for 72 h. (**C**) The expression of phospho-Rb in AY27 cells after Trolox treatment compared to the control cells was measured by WES, N = 8. (**D**) Proliferation curve of AY27 cells cultured in standard media supplemented or not with Trolox, N = 5. (**E**) The number of AY27 cells in each phase of the cell cycle was examined by flow cytometry with propidium iodide DNA staining (N = 5). * *p* < 0.05, ** *p* < 0.01 compared with Ctr cells. All the values are expressed as mean ± SEM.

We then asked whether the effects of MAOs were linked to the generation of ROS. Overactivation of MAOs can lead to the generation of H_2_O_2_ in proximity to the mitochondria, as previously demonstrated in cardiac cells [9]. We first evaluated the levels of total or mitochondrial ROS in AY27 cells in the presence of the MAO inhibitor pargyline. As shown in Figure 4A, total levels of ROS were not modulated by MAO inhibition. On the other hand, mitochondrial H_2_O_2_ levels, measured with the mitochondria-directed peroxy-yellow fluorescent probe (mitoPY1), were significantly decreased with pargyline (Figure 4B). As expected, the antioxidant Trolox strongly reduced both total and mitochondrial ROS levels (Figure 4A,B). These results indicate that the chronic activation of MAOs in AY27 cells promotes an intra-mitochondrial build-up of H_2_O_2_. We next evaluated whether antioxidant treatment had any effect on the progression of the cell cycle by measuring the phosphorylation of Rb in AY27 cells. We found that phospho-Rb was significantly decreased in the presence of Trolox, confirming that ROS were important for the control of the cell cycle in AY27 cells (Figure 4C). As a consequence, Trolox inhibited the proliferation of AY27 cells (Figure 4D) and blocked the cell cycle in the G1 phase, leading to a reduced number of cells in the G2/M phase (Figure 4E).

**Figure 5:**
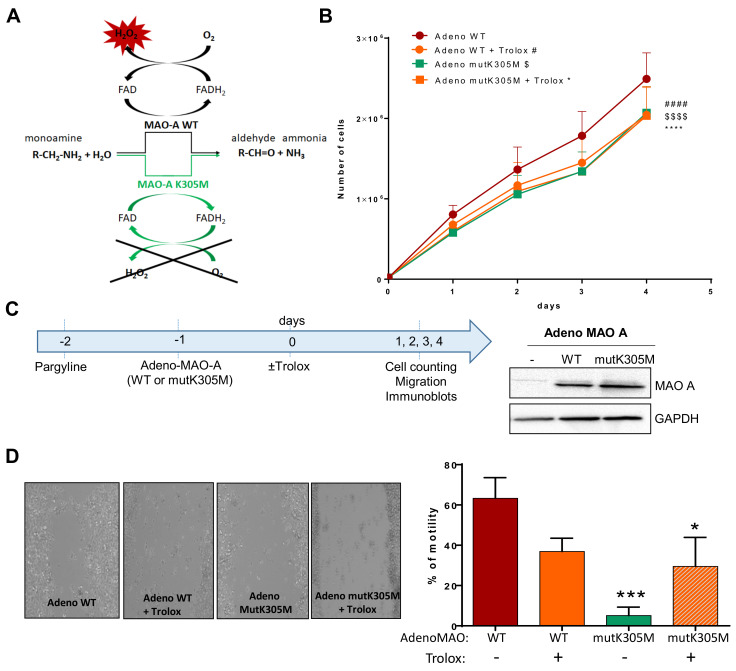
Effects of WT or K305M MAO-A in AY27 tumorigenesis. (**A**) MAO-A K305M mutant construct is unable to use O_2_ as an electron acceptor for reoxidation of the enzyme, preventing the formation of H_2_O_2_. An alternative electron acceptor is used to recycle the mutant enzyme, which is still able to degrade amine substrates (Iacovino et al., 2020). (**B**) Proliferation curve of AY27 cells after adeno WT or adeno mutK305M transductions. Cells were treated or not with Trolox and counted each day until 96 h (N = 4). (**C**) After pretreatment with pargyline to block endogenous MAO activity, cells were transduced or not (Ctr) with adenovirus carrying either the wild-type (WT) or mutant K305M (mutK305M) forms of MAO-A. Western blot analyses were performed at 72 h post-transduction (N = 3). (**D**) AY27 cells transduced with adeno WT and adeno mutK305M were treated or not with Trolox, wounded and then re-treated for 48 h (N = 4). * *p* < 0.05, *** *p* < 0.01, #### *p* < 0.0001, $$$$ *p* < 0.0001 compared with adeno WT. All the values are expressed as mean ± SEM.

As Trolox is a global antioxidant that inhibits ROS from all cell sources, we next aimed at evaluating the specific role of mitoROS generated by MAO-A in the proliferation of AY27 cells. To reach this goal, cells were transduced with an adenovirus expressing recombinant human MAO-A, either WT or mutated on lysine 305 (K305M). The K305M MAO-A mutant targets an amino acid (Lys305) playing a key role in O_2_ reactivity, leading to H_2_O_2_ production (Figure 5A). This K305M variant of MAO-A has been previously shown to display similar enzymatic turnover of substrate without ROS generation in vitro [21]. In order to eliminate the interference from endogenous MAO enzymes, we first incubated AY27 cells with the irreversible non-selective inhibitor pargyline for 24 h and then washed them with PBS and transduced them the adenovirus (Figure 5C, left panel). Both WT and the K305M MAO-A mutant were expressed at similar levels after transduction in AY27 cells (Figure 5C, right panel). Most interestingly, we observed that cells expressing the K305M MAO-A mutant proliferated significantly less than the cells expressing WT MAO-A (Figure 4B). This demonstrates that H_2_O_2_ production is an important determinant of cell proliferation induced by MAO-A. In order to confirm the role of ROS, we incubated the transduced cells with Trolox during the time course of the experiment. We found that Trolox significantly reduced the proliferation of AY27 cells transduced with WT MAO-A to the same level as AY27 cells transduced with K305M MAO-A, which are deficient in H_2_O_2_ production (Figure 5B). Next, we evaluated the effects of WT and K305M MAO recombinant proteins on motility by wound healing assay. While MAO-A WT-transduced cells were able to repopulate the scratch area at 60% extent, K305M-transduced cells were completely deficient (Figure 5D). As observed before, the antioxidant Trolox reduced cell motility of AY27, confirming the importance of ROS in this process (Figure 5D). In conclusion, the generation of H_2_O_2_ by MAOs is essential for tumorigenesis of bladder cancer cells.

### 2.4. Metabolic Signature in Human Bladder Tumors and Role of MAOs in Glucose Utilization

**Figure 6:**
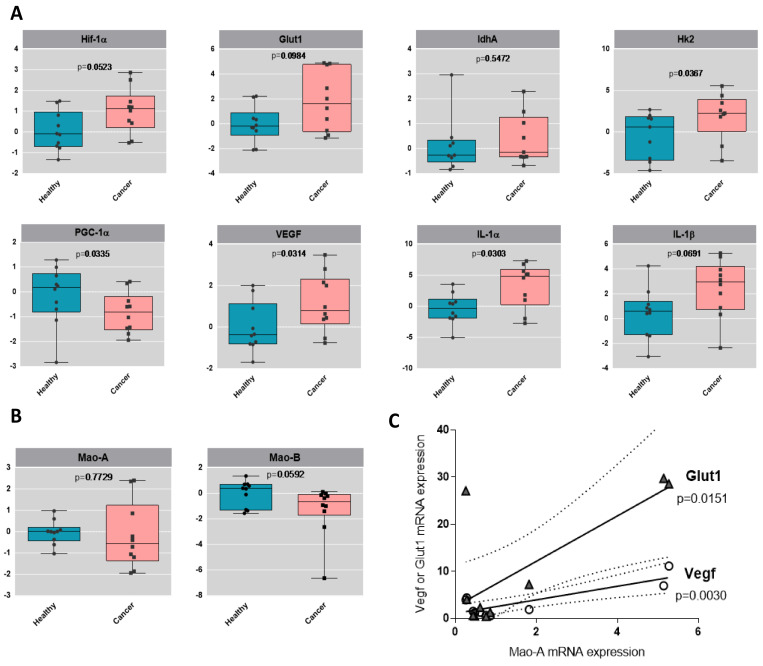
Glycolytic profile of human bladder tumors. (**A**) Boxplots of the gene expression by RT-qPCR analysis of HIF-1α, GLUT1, LDHA, HK2, PGC-1α, IL-1α, IL-1β and VEGF in healthy human samples and in cancer human samples, N = 9. Values are log2 transformed and *p*-values from t-test are displayed. (**B**) Boxplots of the gene expression by RT-qPCR analysis of MAO-A and MAO-B in healthy human samples and in cancer human samples, N = 9. Values are log2 transformed and *p*-values from t-test are displayed. (**C**) Correlation analysis between MAO-A expression and GLUT1 or VEGF expression levels in the different patients.

Urothelial tumors have been previously demonstrated to be highly glycolytic, a property that is believed to favor their aggressive potential. By analyzing the human bladder tumors, we observed a clear trend toward an increased mRNA level of the glycolytic genes GLUT1 and HK2, compared to their healthy counterparts, while the mitochondrial marker PGC-1α was downregulated (Figure 6A). One canonical mechanism of ROS-mediated upregulation of glycolytic genes is through the HIF-1α master regulator [22]. Indeed, we observed upregulation of HIF-1α in human bladder tumor samples, together with VEGF (angiogenesis) and IL-1α and IL-1β (inflammation), which are indicative of poor prognosis in cancer and aggressiveness [23] (Figure 6A). We also measured the expression of MAO-A and MAO-B, and interestingly, we found a positive correlation between the levels of MAO-A in patients and the expression levels of GLUT1 or VEGF (Figure 6B,C), indicating a possible association of MAO-A with glycolysis and angiogenesis.

**Figure 7:**
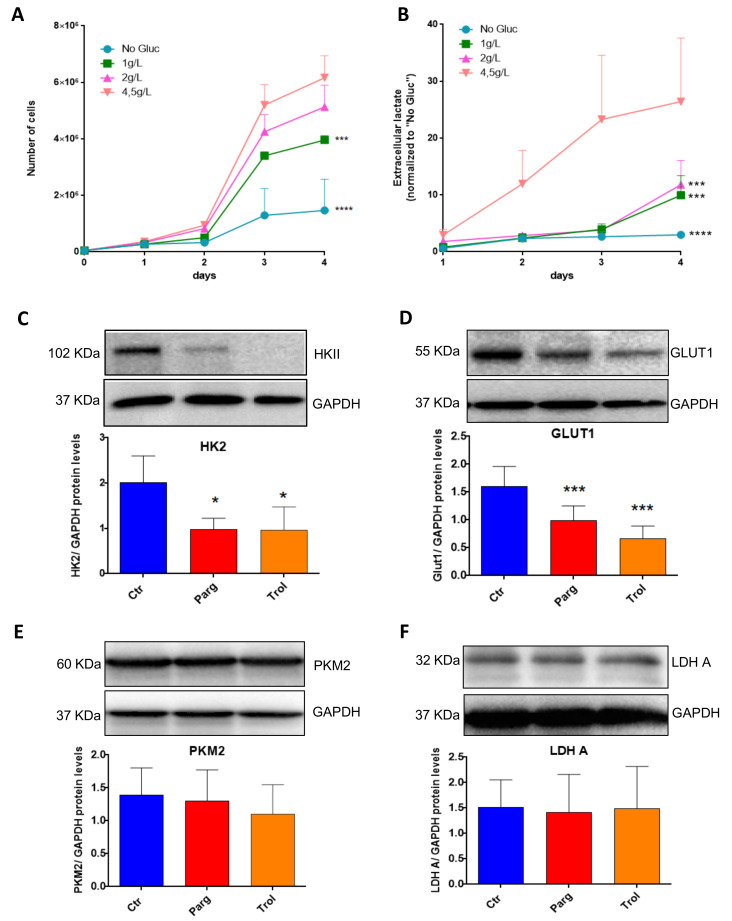
MAOs regulate protein levels of GLUT1 and HKII in AY27 cells. (**A**) Proliferation curves of AY27 cells cultured in the presence of different concentrations of glucose (0 g/L, 1 g/L, 2 g/L, 4.5 g/L). Cells were counted each day until 96 h (N = 3). (**B**) Lactate concentrations in the supernatants of AY27 cells cultured in the presence of different concentrations of glucose (0 g/L, 1 g/L, 2 g/L, 4.5 g/L) for 96 h (N = 3). (**C**–**F**) Western blot analysis of HK2, GLUT1, LDHA and PKM2 protein expression levels in AY27 cells after 96 h of treatment with pargyline or Trolox (N = 5). All the values are expressed as mean ± SEM. * *p* < 0.05, *** *p* < 0.001, **** *p* < 0.0001 compared with No Gluc (**A**,**B**) or Ctr conditions (**C**–**F**).

**Figure 8:**
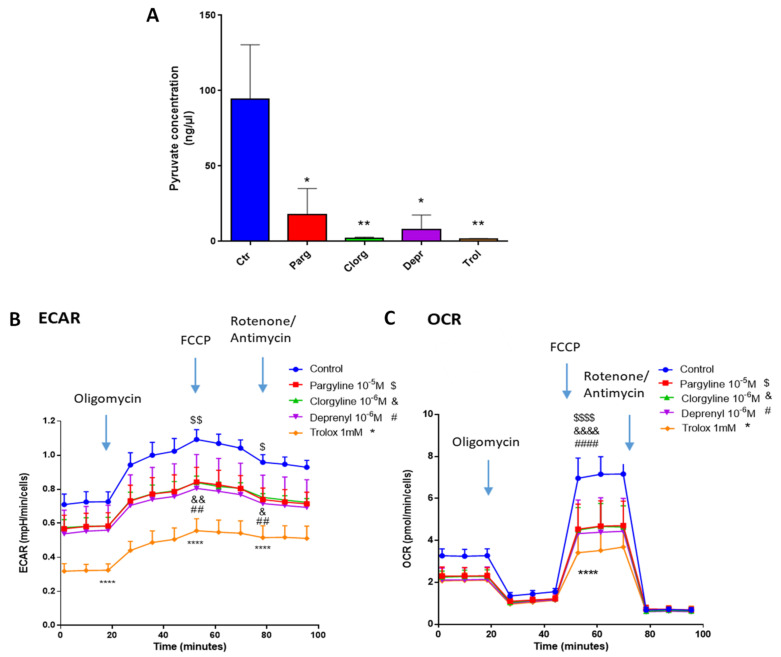
MAOs regulate glycolysis in vitro. (**A**) Pyruvate Assay Kit was used to determine the concentrations of pyruvate in AY27 cells treated with different inhibitors for 96 h (pargyline 10^−5^ M, clorgyline 10^−6^ M, deprenyl 10^−6^ M) and Trolox 1 mM (N = 3). (**B**,**C**) Extracellular acidification rate (ECAR) and oxygen consumption rate (OCR) of AY27 cells cultured with different inhibitors (pargyline 10^−5^ M, clorgyline 10^−6^ M, deprenyl 10^−6^ M) or Trolox were measured with Seahorse XFe24 (N = 3). All the values are presented as mean ± SEM. * *p* < 0.05, ** *p* < 0.01, **** *p* < 0.0001, & *p* < 0.05, && *p* < 0.01, &&&& *p* < 0.0001, ## *p* < 0.001, #### *p* < 0.0001, $ *p* < 0.05, $$ *p* < 0.01, $$$$ *p* < 0.0001 vs. control.

We next assessed whether our in vitro model of AY27 cells displayed the same metabolic features as human bladder tumors. As expected, AY27 cells were highly dependent on glucose since a reduction in glucose in the culture media led to drastic inhibition of their proliferation, together with decreased secretion of lactate, the end-product of glycolysis in the extracellular medium (Figure 7A,B). In order to assess the implication of the MAO/H_2_O_2_ axis in the glycolytic metabolism of AY27 cells, we measured the protein expression of key glycolytic enzymes in the presence of pargyline. Most interestingly, MAO inhibition decreased the expression levels of GLUT1 and HK2, which constitute early steps in glucose transport and transformation, without changes in the expression levels of PKM2 and LDHA (Figure 7C–F). This inhibitory effect was also observed in the presence of Trolox, indicating that MAO-derived ROS tightly regulate the expression of glycolytic enzymes. We next evaluated the intracellular concentration of pyruvate, the end-product of glycolysis that can be either transformed to lactate or used by the mitochondria. As expected, pyruvate content was greatly decreased at 96 h after MAO inhibitor treatments or Trolox, compared to control cells (Figure 8A). This is in agreement with an impairment of glucose transformation and the diminution of GLUT1 and HK2 expression levels by MAO inhibitors. Finally, we evaluated the functional impact of MAO/ROS inhibition on AY27 energetic metabolism. Seahorse analysis revealed that application of pargyline, clorgyline, deprenyl or Trolox potently inhibited glycolysis in AY27 cells, as shown by a decrease in extracellular acidification rate (ECAR) compared to control cells (Figure 8B). This decrease in ECAR is in line with the decrease in intracellular pyruvate in cancer cells, which use glucose as a primary substrate for growth. As many cancer cells retain their capacity to use OXPHOS for the production of ATP, we evaluated the mitochondrial respiration in parallel with Seahorse. Interestingly, oxygen consumption rate (OCR) was also decreased with MAO inhibitors and Trolox (Figure 8C), which is consistent with the decreased availability of pyruvate.

## 3. Discussion

The results described above strongly support a role for MAOs in bladder cancer tumorigenesis through ROS production and metabolic reprogramming. Importantly, we show for the first time that MAO inhibition reduces two deleterious features of cancer cells, proliferation and migration. While MAOs have been studied for a long time for their roles in neurotransmitter homeostasis and neuropsychiatric diseases, there has been a renewed interest in recent years due to their unexpected function in tumorigenesis and metastasis [5]. A pioneer work performed in 2014 demonstrated for the first time that aggressive prostate cancers had increased MAO-A expression and that knockdown of MAO-A reduced tumor growth and metastasis in vivo through HIF-1α and epithelial-to-mesenchymal transition blockade [12]. Since then, a role for MAOs has been demonstrated in other types of cancer such as lung cancer [10], glioblastoma [11], colorectal cancer [24] or gastric cancer [25]. This has led to the identification of MAOs as new therapeutic targets in cancer, and MAO inhibitors are currently in development to be used in combination with some chemotherapeutic agents [26]. Some clinically approved selective MAO-A (clorgyline) or MAO-B (selegiline, rasagiline) inhibitors and the non-selective inhibitor pargyline are irreversible in nature, which causes significant adverse effects. This is why new reversible MAO inhibitors, devoid of harmful effects, are actively searched for [27]. In addition, some natural products from plant sources with reversible MAO inhibitory properties have been identified and are being tested in different pathological contexts. For example, some alkaloids act as potent MAO-A inhibitors and may provide beneficial effects on neuroblastoma and other cancer cell lines [28].

Our present findings constitute the first demonstration of the biological role of MAOs in the proliferation and migration of rat and human bladder cancer cells. One previous study found increased expression of MAO-A in bladder cancer tissues [29]. However, our experiments on resected tissues from human bladder cancers showed only a trend for increased expression of MAO-A in cancer while MAO-B was not modified. Therefore, while both MAO isoforms are present in the bladder, we cannot conclude that there is an upregulation of their expression levels in cancer. Another way of regulating MAO catalytic activity is through the regulation of substrate availability. Most interestingly, some new pieces of evidence have pointed out the formation of adrenergic nerve fibers that infiltrate and expand in many solid tumors such as prostate, gastric, breast, pancreatic, colon and skin cancers [30]. Although not yet described in the bladder, these findings support the existence of a local source of norepinephrine in the solid tumor microenvironment that could fuel MAO catalytic activity [31].

Concerning the respective roles played by MAO-A and MAO-B in urothelial tumorigenesis, we observed some redundancy since both MAO-A and MAO-B inhibitors blocked proliferation and glycolysis. On the other hand, AY27 cell motility was inhibited only by clorgyline, but this could be due to the higher expression of MAO-A compared to MAO-B in these cells. In the present study, all our findings point toward the crucial role of ROS in MAO-driven activation of tumorigenesis. It is well known that cancer cells have increased ROS levels, although the exact cellular sources are not identified, and that they are capable of coping with enhanced oxidative stress to evade cell death through the upregulation of antioxidant defenses [14]. This profound remodeling of ROS-mediated signaling pathways is essential for the initiation, progression, angiogenesis and metastasis of cancer. By the use of a MAO-A recombinant protein that is unable to produce H_2_O_2_, we found that the effects of MAO-A on proliferation and motility were abolished. This mutant has been previously demonstrated to metabolize MAO substrates, but it does not react with O_2_ to generate H_2_O_2_. Instead, the K305M MAO-A recombinant protein is supposed to use alternative electron acceptors (possibly quinones) in order to reoxidize the enzyme. In cardiac cells, MAO-dependent ROS production is impaired with the MAO-A K305M mutant, together with senescence activation [21]. Using this molecular tool, we clearly demonstrate that ROS production is necessary for MAO-A-dependent tumorigenesis of AY27 cells. In addition, we provide evidence that MAO inhibition decreases mitochondrial ROS levels, using the mitoPY1 probe in AY27 cells, but not total ROS levels (DCFDA), which is consistent with the localization of the enzymes at the mitochondrial outer membrane. In addition, since the mitoPY1 probe is more specific for H_2_O_2_, it is possible that MAO inhibition cannot decrease global ROS, as measured with DCFDA [32]. This points toward the existence of other sources of ROS in AY27 cells that we did not identify in the present study but are part of the complex ROS regulatory mechanisms in cancer cells. Regardless, all these results clearly point toward a role for MAO-derived H_2_O_2_ in tumorigenesis in AY27 cells. On the other hand, the global antioxidant Trolox, which is a vitamin E analog, was even more potent than pargyline in reducing proliferation, motility and glycolysis in AY27 cells. However, Trolox could have secondary effects on ROS signaling in normal cells due to its strong antioxidant properties and lack of specificity. With the failure of global antioxidant therapies in clinical trials, it might be more appropriate to target specific ROS sources in cancer such as MAOs [15].

Most interestingly, one of the major findings of our study is the ability of MAO inhibitors to block glucose utilization in urothelial cancer cells. Metabolic reprogramming, characterized by the upregulation of aerobic glycolysis in cancer cells, constitutes a new target for therapeutic intervention since transformed cells are highly dependent on this pathway to sustain their high level of proliferation [17]. The activation of glycolysis in cancer cells generates lactic acid that impairs the growth of normal cells and facilitates metastasis. In addition, the activation of glycolysis in cancer cells reinforces antioxidant response through enhanced glutathione levels and maintains high levels of glycolytic intermediates to support anabolic reactions [33]. In our study, ex vivo transcript analysis of human bladder cancer tissues demonstrated the upregulation of glycolytic genes GLUT1 and HK2 together with the downregulation of PGC-1α, supporting the presence of a metabolic switch toward glycolysis. In vitro, we indeed observed a strong dependency of AY27 cells toward glucose substrate, as glucose deprivation completely blocked proliferation. In accordance, AY27 cells generate large amounts of lactate, which is released in the extracellular media, meaning that aerobic glycolysis is upregulated. In parallel, we observed that AY27 cells retained some functional mitochondrial respiration, in contrast to “Warburg’s theory” [34]. Indeed, it is now shown that some cancer cells have fully functional OXPHOS machinery and can switch between OXPHOS and aerobic glycolysis, or even do both at the same time, according to the “metabolic flexibility” theory. Here, we show that MAO inhibitors and Trolox decreased glycolysis and OXPHOS, meaning that they strongly impaired glucose-dependent metabolism in AY27 cells. Indeed, MAO inhibitors and Trolox potently decrease the expression of GLUT1 and HK2, which are critical enzymes in the regulation of glucose transport and utilization. On the other hand, we could not find any effect of MAO inhibitors or Trolox on the expression of other glycolytic enzymes such as LDHA and PKM2 in AY27 cells, which supports the idea that the regulation by MAOs occurs in the early steps of glucose utilization. As a consequence of decreased glucose transport, MAO inhibitors strongly reduced pyruvate accumulation in AY27 cells, which could be responsible for the reduction in glycolysis and mitochondrial respiration that we observed. Thus, based on our findings, we believe that MAOs promote the transport of glucose across the membrane and its transformation to facilitate glucose-dependent metabolism which is crucial for proliferation and survival. Upregulation of glucose transporters such as GLUT1 and GLUT3 constitutes a signature of cancer, which is used as a diagnosis with FDG-PET, but this overexpression is also associated with aggressiveness and invasiveness of tumors [35]. Thus, glucose transporter inhibitors are actively explored for their capacity to inhibit tumorigenesis [36]. Interestingly, in cardiac cells, MAO-A activation increased GLUT1 and GLUT4 expression levels, favoring glucose transport through a ROS-mediated mechanism [37], indicating that this might be a more general mechanism of action of MAOs. It will be interesting in the future to decipher whether the regulation of glycolysis by MAOs is a common mechanism in other types of cancer for which MAOs promote tumorigenesis (prostate cancer, glioblastoma, lung cancer).

In prostate cancer, MAO-A appears to specifically modulate EMT, proliferation and metastasis [12]. Indeed, MAO-A knockdown reduces tumor growth and metastasis by inhibiting HIF-1α and EMT in aggressive prostate cancers. In our hands, the application of MAO inhibitors in AY27 cells failed to modulate N-cadherin, E-cadherin and vimentin protein levels. Instead, we observed a strong regulation of VEGF, IL-1α and IL-1β in tumors of patients and a correlation between VEGF and MAO-A mRNA expression levels. In the present study, we did not explore the role of MAOs in the regulation of angiogenesis and inflammation, but this could be very interesting in the future. In conclusion, we identified a new potential therapeutic target in bladder cancer through MAO-dependent ROS inhibition that will need to be tested in combination with classical chemotherapeutic drugs.

## 4. Materials and Methods

### 4.1. Bladder Cancer Cell Lines

The AY27 tumor cell line was initially derived from carcinomas of the urinary bladder induced in male Fischer (F344) rats fed with FANFT (N-[4-(5-nitro-2-furyl)-2-thiazolyl]formamide). AY27 cells were cultured in RPMI-1640 medium (GIBCO, Invitrogen Courtaboeuf, France) supplemented with 10% FBS (Biotech, GmbH, Rheinbrohl, germany), 5% L-glutamine (200 mM) and 1% penicillin/streptomycin. Cells were maintained at 37 °C in a humidified incubator with 5% CO_2_. *T*24 cells were seeded in DMEM medium supplemented with 5% FBS, 15 mM HEPES, 2 mM L-glutamine, 1% insulin/transferrin/selenium and 1% penicillin/streptomycin.

### 4.2. Human Tissue Collection and Ethics

Urinary bladder specimens were obtained from 10 patients with characteristics described in Table 1.

Patients had undergone cystoprostatectomy or pelvectomy for bladder cancer in the Urology Department of Rangueil Hospital (Toulouse, France) and provided written informed consent prior to inclusion in the study. The current study was conducted according to the European Council Directive 2006/17/CE regarding technical requirements for the donation, procurement and testing of human tissues and cells. Activities of preparation, use and exportation of human tissues are authorized for Urosphere by French Authorities (Declaration/Authorization CODECOH No. DC-2019-3563/AC-2019-3565). After sampling, the tissues were frozen in liquid nitrogen and stored at −80 °C.

### 4.3. RNA Isolation, cDNA Synthesis and Quantitative Polymerase Chain Reaction (qPCR)

Total RNA from human tissues was isolated with RNeasy Kit (RNeasy Mini Kit, QIAGEN), following the manufacturer’s instructions, and quantified by spectrophotometry (ND-100 NanoDrop, Thermo Fisher Scientific, Illkirch-Graffenstaden, France). cDNA was synthesized with the iScript cDNA Synthesis Kit (BIO-RAD) according to the manufacturer’s instructions. PCR was performed in triplicate for 40 cycles with SYBR green (Takara, Ozyme) on a QS5 thermocycler (Thermo Fisher Scientific). Primers were designed and checked using Primer-BLAST and are listed in Supplementary Table S1. β-actin was used as the reference gene for normalization. Results are expressed as relative gene expression compared to the housekeeping gene (2^−∆*C*t^) and compared to the control group (2^−∆∆*C*t^), calculated using the comparative cycle threshold (Ct) method.

### 4.4. Western Blotting

Total protein extracts of human tissues or cells were lysed in RIPA buffer (50 mM Tris pH 7.2, 500 mM NaCl, 1% Triton X100, 1 mM EDTA, 100 mM sodium fluoride, 5 mM sodium orthovanadate and 10 mM sodium pyrophosphate) and centrifuged for 15 min at 17,000× *g*. The supernatant was taken and the proteins were quantified using the BCA kit (Sigma-Aldrich, Saint-Quentin-Fallavier, France). Equal amounts of proteins were separated by electrophoresis on SDS-polyacrylamide gel (80 mV/1.5 h) and then transferred to a nitrocellulose membrane (Millipore) in a tank blot apparatus (Bio-Rad Laboratories). Membranes were blocked in Tris-buffered saline (TBS) solution containing 3% BSA and 0.01% Tween (TBS-T) for 1 h at RT and incubated with the corresponding primary antibodies diluted in 3% BSA-TBST overnight at 4 °C (Supplementary Table S2). The next day, membranes were incubated with HRP-conjugated antibodies, and antigen–antibody complexes were detected using Super Signal Chemiluminescent Substrate (ECL Pierce). Images were acquired using the Chemidoc-MP imaging system and quantified using Image Lab 6.0 software (Bio-Rad). For phospho-Rb (Ser807/811) (Cell Signaling Technology #9308), protein quantification was performed using a capillary-based “Simple Western” system (Jess, ProteinSimple, bio-techne, Noyal Châtillon sur Seiche, France). Separation and immunoprobing were performed automatically, and the chemiluminescent signals were detected and analyzed using Compass software (ProteinSimple, bio-techne, Noyal Châtillon sur Seiche, France). Total protein normalization was achieved with total protein HRP labeling, and total protein value was fully analyzed by the software.

### 4.5. Proliferation Assay

Cells were plated at a density of 40,000 cells per well. After 24 h, cells were treated for 4 days with pargyline 10^−5^ M, clorgyline 10^−6^ M, deprenyl 10^−6^ M or Trolox 1 mM, and/or transduced with adenovirus expressing MAO-A WT or MAO-A K305M mutant [21]. For this part of the protocol, the cells were pretreated with pargyline for 24 h in order to block the endogenous MAOs, and then the adenovirus transduction was performed. Cells were either manually counted each day for 4 days (AY27 cells) or automatically counted every 2 h (T24 cells) using the Incucyte live-cell imaging system (Sartorius, Dourdan, France).

### 4.6. Motility Assay

Thirty thousand cells were plated per well and treated for 48 h with MAO inhibitors pargyline 10^−5^ M, clorgyline 10^−6^ M, deprenyl 10^−6^ M or Trolox 1 mM and/or transduced with adenovirus expressing MAO-A WT or MAO-A K305M mutant [21]. For this part of the protocol, the cells were pretreated with pargyline for 24 h in order to block the endogenous MAOs, and then the adenovirus transduction was performed. Upon reaching confluence, the cells in the monolayer were vertically scratched using a pipette tip. Images were captured every 30 min over a 24 h period using a video microscope to observe cell motility (wound closure). Images were individually analyzed using image analysis software, and the percent wound closure area was calculated at 24 h.

### 4.7. Cell Cycle Assay

Cells were fixed and permeabilized with ethanol 70% on ice. After centrifugation, the pellet was incubated with a solution containing RNAse 1 mg/mL and propidium iodide (PI) 0.4 mg/mL for 30 min at 37 °C. Cells were gently resuspended and passed through a 40 µm filter to remove aggregates. Samples were analyzed on a BD FACS Verse flow cytometer. After appropriate FSC vs. SSC gates were used to exclude debris and cell aggregates, the PI histograms were analyzed with a cell cycle plug-in in FlowJo V10.8 software (BD Biosciences) using the Dean–Jett–Fox model.

### 4.8. Monoamine Oxidase Activity

The MAO-Glo Assay kit (Promega, Madison, WI, USA) was used according to the manufacturer’s protocol. Human samples and approximately 4 million cells were incubated with MAO inhibitors for 15 min. After the addition of luminogenic MAOs substrate, MAO-A and MAO-B activities were assayed in their respective buffers and the luminescence was measured.

### 4.9. ROS Measurements

Cells were plated and treated with pargyline or Trolox for 72 h. After that, the medium was replaced with HBSS for 2 h. For mitochondrial H_2_O_2_ measurements, cells were incubated with the mitoPY1 probe (Mitochondria Peroxy Yellow 1, Sigma) at a final concentration of 10 μM at 37 °C for 60 min. For total cellular ROS, cells were incubated with DCFDA probe at a concentration of 5 µM at 37 °C for 60 min (Thermo Fisher Scientific). Cells were collected and fluorescence was detected using the GFP channel on a BD FACS Verse flow cytometer.

### 4.10. Mitochondrial Respiration

The oxygen consumption rate (OCR) was measured with a Seahorse XFe24 Analyzer (Agilent). Cells were plated at a density of 1 × 10^5^ cells/well in Seahorse 24-well assay plates in complete medium. After 24 h, the medium was replaced and cells were incubated with pargyline 10^−5^ M, clorgylin 10^−6^ M, deprenyl 10^−6^ M or Trolox 1 mM. After 48 h of treatment, the medium was replaced with XF base medium supplemented with 10 mM glucose, 4 mM l-glutamine and 1 mM sodium pyruvate (pH 7.4). The plates were incubated for an additional 1 h at 37 °C in a non-CO_2_ incubator. The wells of a hydrated sensor cartridge were then loaded with 1 µM oligomycin (port A), 1 µM carbonyl cyanide 4-(trifluoromethoxy)phenylhydrazone (FCCP) (port B) and 1 µM antimycin A + 1 µM rotenone (port C). Data were analyzed using the Seahorse Wave software (Agilent, Les Ulis, France).

### 4.11. Measurement of Lactate

Forty thousand cells were plated per well. After 24 h, cells were incubated for 4 days with basal media without glucose, glucose 1 g/L, glucose 2 g/L or glucose 4.5 g/L. Cells were stopped and counted each day, and for the remaining cells, the medium was renewed. The quantity of lactate released in the extracellular media was measured on different days. For this assay, Lactate Assay Kit (Sigma) was used, and lactate concentration was determined by an enzymatic assay that results in a colorimetric (570 nm)/fluorometric (λex = 535 nm/λem = 587 nm) product, proportional to the lactate present.

### 4.12. Pyruvate Concentration

The pyruvate assay kit (Sigma) was used according to the manufacturer’s protocol. Cells were treated with MAO inhibitors and Trolox for 96 h. After that, pyruvate concentration was determined by a coupled enzyme assay, which results in a colorimetric (570 nm)/fluorometric (λex = 535/λem = 587 nm) product.

### 4.13. Statistical Analysis

Statistical analysis was carried out using Student’s *t*-test, 1-way ANOVA or 2-way ANOVA when appropriate. ANOVA analysis was followed by the Dunnett post hoc test. Values of *p* < 0.05 were considered to be significant.

## Figures and Tables

**Table 1:** **Patient characteristics.** UC, urothelial carcinoma; CIS, carcinoma in Situ.

N° Patient	Sex	Age	Site	Grade	Pathological Stage	Treatment
1	Man	83	Primitive	pT3apN2	UC, infiltrating muscle	Surgery
2	Man	56	Primitive	pT2pN0M0	UC, infiltrating muscle	Chemotherapy
3	Woman	72	Recidive	pT2b	UC	Surgery
4	Man	61	Primitive	pT2pN0M0	CIS	Surgery
5	Man	71	X	pTa	UC, not infiltrating. High grade	Surgery
6	Man	60	X	pT2bNxMx	UC, infiltrating muscle	Chemotherapy
7	Man	64	X	X	UC, infiltrating muscle	Chemotherapy
8	Man	75	Recidive	pT3pNxMx	UC, infiltrating muscle	Surgery
9	Man	58	X	x	x	Chemotherapy
10	Man	61	x	pT2	UC, infiltrating muscle	Chemotherapy

## Data Availability

The data presented in this study are available on request to the corresponding author.

## Supplementary Materials

The following supporting information can be downloaded at: https://www.mdpi.com/article/10.3390/ijms231911747/s1.
