# Supplementary material for: Monoamine Oxidase Inhibitors Prevent Glucose-Dependent Energy Production, Proliferation and Migration of Bladder Carcinoma Cells
Source: Int J Mol Sci. 2022 Oct 4;23(19):11747. doi: 10.3390/ijms231911747 (PMC9570004; doi:10.3390/ijms231911747)
Supplement: Supplementary file 1 [file ijms-23-11747-s001.zip › ijms-1858167-supplementary.pdf]

Supplementary material and methods

Annexin-V-FITC and propidium iodide staining. AY27 cells were seeded in six-well plate (1×10^5cells per well). After 24 h, cells were treated with Pargyline 10^{-5}M for 48 h and then harvested for co-staining with FITC Annexin V Apoptosis Detection Kit I (BD Pharmingen, San Jose, CA, USA) following instructions of the manufacturer. Analyses performed on samples were analyzed on BD FACS Verse flow cytometer, use 488 nm excitation and emission collected at 530 nm (FITC) and 575–610 nm (PI). After an appropriate FSC vs. SSC gates to exclude debris and cell aggregates, in BD FACSuite software (BD Biosciences). With bivariant dot plot, we distinguish between viable cells (Annexin V− / PI−), early apoptotic cells (Annexin V+ / PI−), late apoptotic/necrotic cells (Annexin V+ / PI+) and late necrotic cells (Annexin V− / PI+).

SA-βgal assay. Cells fixed in PFA were stained with SA-β-gal staining solution (1 mg/ml X-gal; 40 mM citric acid/sodium phosphate pH 6; 5 mM potassium ferrocyanide; 5 mM potassium ferricyanide; 150 mM NaCl; 2 mM MgCl2). After 16 h at 37°C, cells were counterstained with hematoxillin and images were taken using a brightfield microscope.

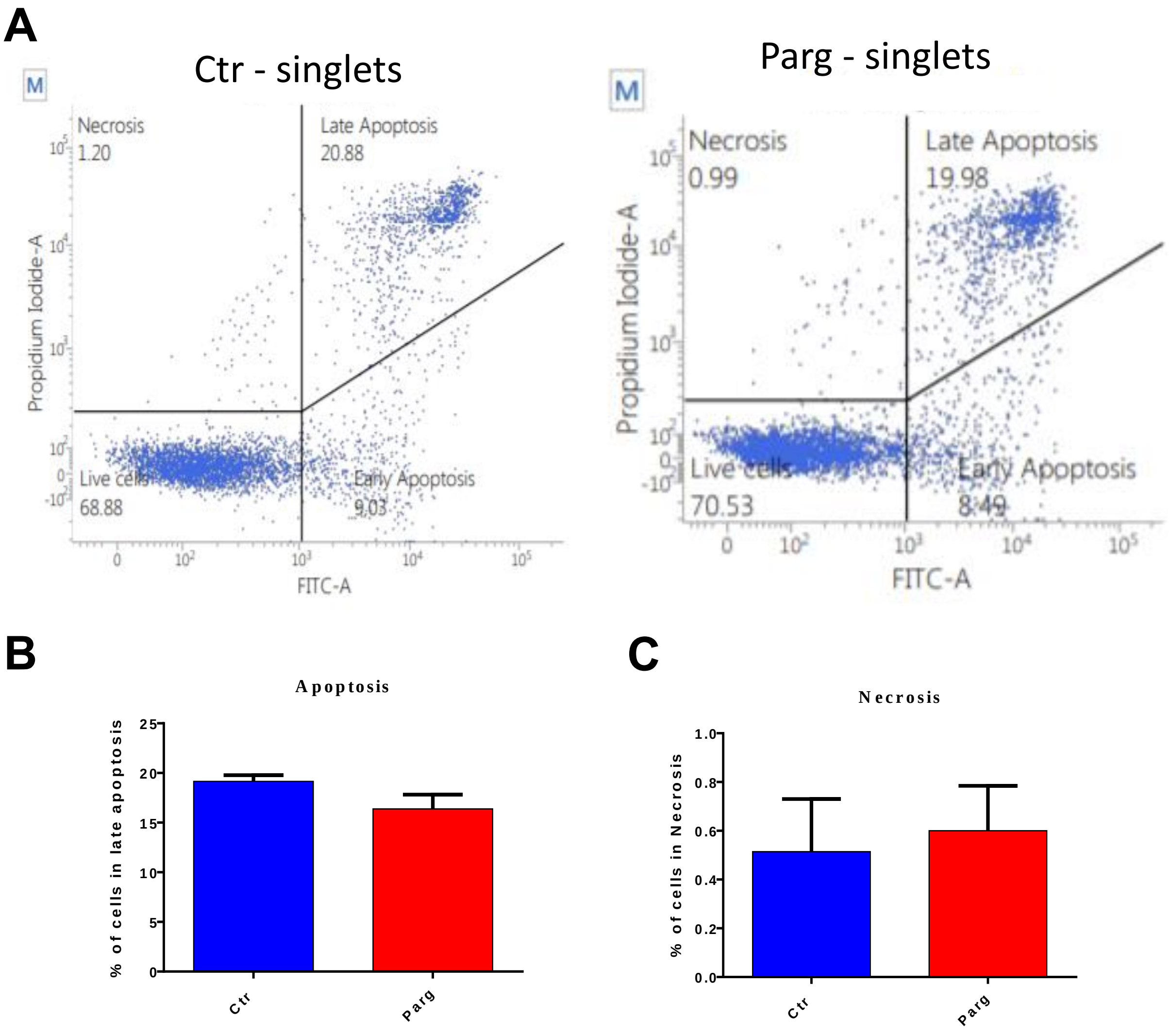

Suppl Figure S1. Apoptosis and necrosis measurements. AY27 cells were treated with Pargyline 10^{-5}M for 48 h. Apoptosis and necrosis were measured by flow cytometry analysis after propidium iodide and Annexin-V-FITC labelling. **(A)** Flow cytometry dot-plot from a representative experiment. **(B)** Histogram representing the % of apoptotic cells population (Annexin-V-positive cells $^{+}$Annexin-V/propidium iodide-positive cells) for each condition. N=4. **(C)** Histogram representing the % of necrotic cells population (Annexin-V-positive cells^{+} Annexin-V/propidium iodide-positive cells) for each condition. Values are presented as the means ± SEM (N=4).

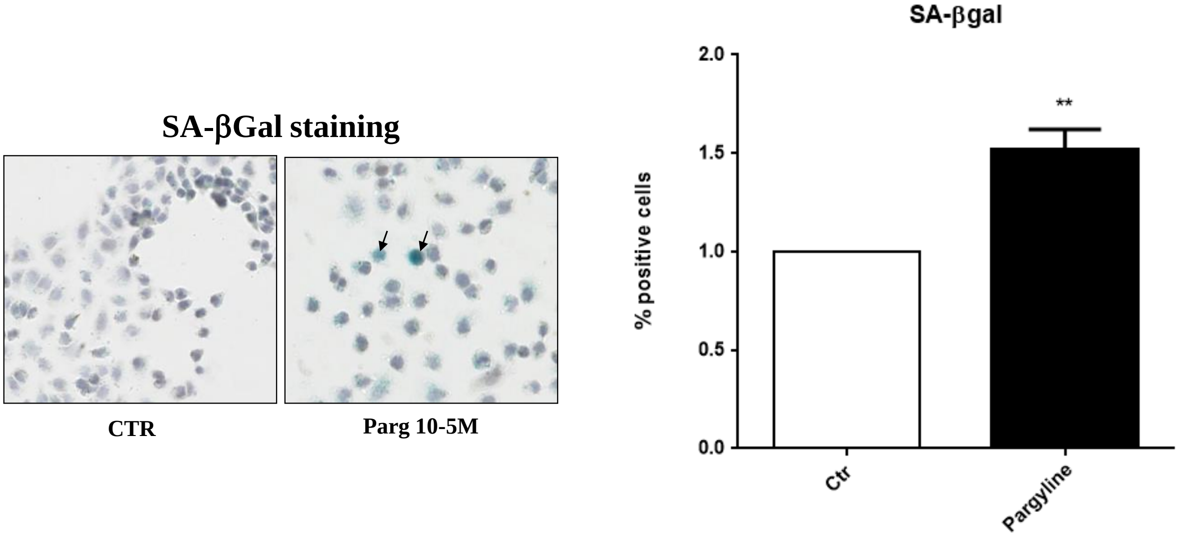

Suppl Figure S2. Senescence measurements. AY27 cells were treated with Pargyline 10^{-5}M for 96 h and SA-βgal activity was measured. SA-βgal-positive cells are stained blue and expressed as percent of total cells. Values are presented as the means ± SEM (N=4). **p<0.01

Supplementary Table S1: list of primers for QPCR

Gene Name	Primer Name	Sequences 5' → 3'
Hif1α	Hif1α fw	CAGAGCAGGAAAAGGAGTCA
	Hif1α rv	AGTAGCTGCATGATCGTCTC
Slc2a1	Glut1 fw	GTCACCATCCTGGAGCTGTT
	Glut1 rv	GAAGGCCGTGTTGACGATAC
Ldha	LdhA fw	GATCTCATTGCCACGCGCC
	LdhA rv	TCAGCTGATCCTTTAGAGTTGCCA
Hk2	Hk2 fw	AAATCAGCCTCGGGACCGC
	Hk2 rv	CAGAGCCAGGAACTCTCCGT
Ppargc1a	Pgc1α fw	TGACTGGCGTCATTCAGGAG
	Pgc1α rv	CCAGAGCAGCACACTCGAT
Il1a	Il1α fw	TGTATGTGACTGCCCAAGATGAAG
	Il1α rv	AGAGGAGGTTGGTCTCACTACC
Il1b	Il1β fw	TCAAGTGTCTGAAGCAGCCAT
	Il1β rv	GTCCTGGAAGGAGCACTTCAT
vegfa	Vegf fw	GCTACTGCCATCCAATCGAG
	Vegf rv	GTCTCCTATGTGCTGGCCTT
Actb	β-actin fw	ATGATGATATCGCCGCGCTC
	β-actin rv	CCACCATCACGCCCTGG

Supplementary Table S2: list of antibodies for immunoblots

Target	Company	Reference
MAO-A	Abcam	126751
MAO-B	Abcam	175136
p-Rb	Cell Signaling	9308S
GLUT1	Cell Signaling	12939
HK 2	Cell Signaling	2867
GAPDH	Cell Signaling	2118S
